# Interaction between prostate cancer stem cells and bone microenvironment regulates prostate cancer bone metastasis and treatment resistance

**DOI:** 10.7150/jca.73143

**Published:** 2022-06-13

**Authors:** Lu Yao, Xiangyu Zhang

**Affiliations:** 1 Department of Clinical Medicine, Jining Medical University, Jining 272067, China.; 2 Department of Pathology, Jining First People's Hospital, Jining Medical University, Jining 272000, China.

**Keywords:** Prostate cancer stem cell, CRPC, Bone microenvironment, Metastasis

## Abstract

Prostate cancer (PCa) is one of the most common cancers with increasing rates of incidence. Bone metastasis and drug resistance are the most serious threat faced by patients following a delayed diagnosis of cancer, which might lead to treatment failure and death. The theoretical model of cancer stem cells (CSCs) explains the diverse molecular characteristics of cancer as well as its relapse, metastasis and drug resistance. Prostate cancer involves heterogeneous cells community, including prostate cancer stem cells as an important component. These subtypes of cancer cells are usually monoclonal, expressing specific biomarkers and exhibiting self-renewal and differentiation capacity. Therefore, therapies that target CSCs might be more effective in overcome drug resistance and metastasis. Thus, anti-CSCs therapies differ from the traditional anti-proliferative approach. We focus here on reviewing the effects of prostate CSCs on bone metastasis and resistance to traditional treatment in PCa, and report new clinical strategies that address CSC-based tumorigenesis.

## Introduction

Prostate cancer (PCa) is the second most common cancer and the fifth most common cause of cancer-related mortality among men worldwide 1, 2. The five-year survival rate in patients with non-metastatic PCa has been consistently growing in recent years, practically approaching 100%. However, advanced PCa often leads to bone metastases, which is currently incurable 3, 4. Skeletal-related events (SREs) due to PCa bone metastasis often cause pain, which reduces the patients' quality of life. Agents that inhibit osteoclastic activity, including zoledronate and denosumab, are usually used clinically to treat patients with PCa metastatic disease 5.

Early-stage PCa is androgen-dependent and surgical prostate removal is the most common treatment strategy, which can effectively cure localized prostate cancer 6. However, most patients with PCa eventually become androgen-independent and progress to castration-resistant prostate cancer (CRPC) 7,8. Castration-resistant prostate cancer usually leads to bone metastasis, which is lethal as no effective treatment currently exists 9-11. Most currently available therapies usually focus on cancer cells in bone metastasis, rather than the bone microenvironment (BME), which plays an important role in prostate cancer bone metastasis. This might be the limitation of current therapy. It is exciting, however, to know that studies have reported the interactions between cancer cells (including CSCs) and BME 12-15. CSCs are an important component of prostate cancer cells and usually promote bone metastasis progression. CSCs exhibit high clonogenic activity and potential for cancer initiation. Accordingly, effective treatment of metastatic PCa requires elucidation of the mechanisms underlying the growth of PCa cells in the BME, especially in the context of the important role played by prostate CSCs (PCSCs). This may shed some light on understanding of prostate cancer bone metastasis and pharmacological target selection when treating this disease.

## Stem cells

Advances leading to the discovery of hematopoietic stem cells have increased the number of treatment options for cancer 16. Among cancer cells, CSCs form a small subpopulation characterized by self-renewal, quiescence, and potential for differentiation, with a key role in cancer recurrence, metastasis, drug resistance and heterogeneity 17-19. CSCs exhibit two types of cell division: 1) symmetric cell division in which one stem cell divides into two stem cells; 2) asymmetric cell division, in which one stem cell forms a new stem cell and a daughter cell 20,21. Adult stem cells are often in a quiescent state, and are regulated by multiple cell cycle regulatory genes, such as *p21*, *p18*, and *p63*. When they exit the quiescent state, these cells self-renew and differentiate into other types cells 22. Bonnet and Dick first demonstrated in 1977 that human acute myeloid leukemia originates in CSCs, which express the cell surface markers CD44^+^ and CD38^-^ 23.

The human prostate is composed of stromal cells and three types of epithelial cells: basal cells, luminal secretory cells, and neuroendocrine cells 24. Only a small subset of basal cells comply with the stem cell hypothesis 25. They can replenish the apoptotic and dead cells. They also induce the continuous differentiation from primitive basal cell to secretory cell. Prostate stem cells are most likely located in the basal layer as a component of the prostate basal cell subpopulation, probably in specific niches. One study indicated that the stem cells express the Zeb1 marker 25, 26. These niches are surrounded by several types of cells, such as mesenchymal stem cells, inflammatory cells, and immune cells. The stromal cells interact with the normal prostate stem cells via multiple biological signals exhibiting paracrine signaling pattern 27, 28. The prostate CSCs may originate in the normal stem cell of prostate via genetic alternations.

## Prostate cancer stem cells

Prostate cancer is a highly heterogeneous entity, comprised of multiple cancer cell subpopulations (including CSCs) and non-tumoral cells such as epithelial cells, stromal cells, immune cells, vascular endothelial cells, tumor-associated macrophages (TAMs) and cancer associated fibroblasts 29-31. Studies involving CSCs has progressed rapidly, and novel techniques for identification, purification, and characterization of these cells have been developed. It is possible that CSCs are derived from normal stem cells, as they display many phenotypic and functional characteristics of normal prostate stem cells. They express several types of cell surface pluripotency makers, including cell-adhesion molecules (CD24, CD44, CD133), transcription factors (OCT4, SOX2, KLF4, NANOG, c-Myc, and HER2), integrin α2β1, and aldehyde dehydrogenase 32-39. Prostate CSCs were found to be innately chemo-resistant and highly metastatic, resulting in clinical recurrence, progression to metastatic disease, and cancer-related death in patients diagnosed with prostate cancer 40. As some CSCs markers are also expressed by normal stem cells, it is necessary to use a combination of several markers to reliably isolate the PCSCs and quantify them. BRCA1 and EZH2 tightly cooperate to regulate the PCSCs phenotype and properties 41. The number of cells expressing ALDH^hi^, CD44^+^, and integrin α2β1^+^ increases when castration resistance occurs, and such cells display potential self-renewal capacity and colony formation, which indicates that castration resistance increase the number of PCSCs 42. Meanwhile, cells expressing CD44^+^, integrin α2β1^+^, and CD133^+^ were isolated from PCa patients, and shown to exhibit self-renewal, suggesting that the three markers can be used to define the CSCs phenotype 43. The use of multiple markers to isolate and characterize CSCs has been widely used; however, some studies propose that the stemness-related transcription factors are also important to identify CSCs.

It is necessary to understand the molecular basis of CSCs stemness, and the mechanism of reprogramming non-CSCs into CSCs. The important transcription regulators, SOX2, OCT4, and NANOG, contribute to the maintenance of the pluripotent state of CSCs. Some studies indicated that inhibition of androgen receptor (AR) function increases the stemness of PCSCs 44, 45. MDM2 mediates AR degradation to maintain the pluripotency of PCSCs. AR alternative splice variants (AR-Vs) are involved in the progression of prostate cancer bone metastasis. AR-Vs also play a role in the resistance to anti-androgen therapy and radiotherapy. The expression of AR-Vs (including AR-V7, AR-V1, and V567es) increased significantly in CRPC bone metastasis when compared with hormone-naïve prostate cancer. AR-V7 induces prostate cancer cell EMT and impart the PCSCs characteristics to prostate cancer cell. NANOG regulates the pluripotency of cancer cells and plays an important role in the evolution of PCSCs. The *SPOP* gene interferes with this process by degrading the NANOG protein. It was inferred that tumor suppressor genes also play an important role in the evolution of PCSCs. Mutations involving genes such as *TP53* and *PTEN* usually occur in PCSCs 46. It is well-known that *BRCA1* and *BRCA2* are tumor suppressor genes, while the mutant genotypes are closely related to different cancer types, including breast cancer and PCa 47, 48. The main role of BRCA1 and BRCA2 proteins is to repair damaged DNA, especially double strand DNA breaks. BRCA1 also plays an important role in maintaining the breast CSC population. BRCA1 and EZH2 cooperate tightly in regulating PCSCs phenotype and properties, and the loss of BRCA1 is related to PCSCs phenotype 41, 49.

## Bone microenvironment of metastatic prostate cancer

Bone is composed of cortical bone and bone marrow in different proportions 50, with the cortical bone surrounding the bone marrow. Cortical bone is highly mineralized when compared with the trabecular bone, and the main mineral substance of bone is hydroxylapatite. Bone extracellular matrix is composed of organic and inorganic components, with collagen constituting the initial architecture, followed by mineralization of apatite. Bone is in a constant process of remodeling, which is mediated by osteoclasts and osteoblasts; osteoblasts promote bone formation while osteoclast promote bone resorption 51-55. Most importantly, many growth factors are found in the bone matrix, including insulin-like growth factors (IGFs), bone morphogenetic proteins (BMPs), transforming growth factor-beta 1 (TGF-β1), and platelet-derived growth factors (PDGFs) 56-59. The bone cells, bone matrix components, and growth factors plays an important roles during PCa metastasis. When prostate cancer cell metastases to the bone, the cancer cell secrete matrix metalloproteinase-9 (MMP-9), urokinase plasminogen activator receptor (uPAR), and cathepsin B (CB), which degrade the bone matrix to release growth factors. Additionally, TGF-β promote bone metastasis via regualtion of prostate cancer cell proliferation, migration and invasion. The Smad protein is phosphorylated and translocated to nucleus after TGF-β stimulation. Interferon-inducible Transmembrane Protein 3 (IFITM3) interacts with Smad 4 to enhance Smad 2 phosphorylation. TGF-β induces EMT and MMP secretion to promote bone mtastasis. TGF-β also activates the Smad-independent pathway, such as the MAPK pathway. Inhibition of TGF-β signaling can attenulating prostate cancer bone mtastasis. TGF-β can also induce immune suppression by regulating immune cell proliferation and differentiation. It can activate the M2 macrophage and inhibit the generation of antigen-presenting dendritic cells. It can also convert neutrophils from CD11b^+^/Ly6G^+^ tumor-associated types to pro-tumor types. As for lymphocytes, TGF-β signaling inhibits the generation of effector T lymphocytes and promote Treg production. It can inhibit B lymphocyte maturation and natural killer cells via mTOR signaling inhibition. Myeloid-derived suppressor cells (MDSCs) can suppress the anti-tumor immunity by inhibiting T cell functions. TGF-β activation can recruit the MDSCs to the prostate cancer microenvironment.

The phenotypic heterogeneity of metastatic prostate cancer cell is established. CSCs are one important component of bone metastasis, and promote bone metastasis progression via various mechanism. Most cancer-related deaths are attributed to metastasis, and PCa is likely to lead to bone metastasis, with devastating consequences for patients' health 60. Several mechanisms play an important role in this process, including epithelial-mesenchymal transition (EMT), during which the CSCs express several EMT markers (vimentin, fibronectin, N-cadherin, and E-cadherin) 61-63. The bone metastatic process mainly involves EMT, dissemination and colonization to bone. EMT plays an important role in initiating bone metastasis. Several signaling molecules, released by the tumor microenvironment, might influence the EMT in PCSCs through autocrine and paracrine signaling manners. These signaling molecules include transforming growth factor β (TGF-β), fibroblast growth factor (FGF), interleukin 6 (IL-6), and hypoxia-induced factor (HIF).

Circulating tumor cells (CTCs) have been known for 150 years, since they were first reported by Ashworth in 1869 64. The hypothesis of “seed and soil” is an important theory of cancer metastasis, in which the CTCs are the “seeds” and the source of distant metastasis 65-67. The EMT process plays a role in regulating entry of the CTCs into the circulation. CTCs contain various types of cancer cells, and the CSCs express specific markers and play a key role in metastasis. Upon reaching the bone via blood vessels after bloodstream circulation, they colonize this site and form niches 68. The bone stromal cells regulate the CSCs, and induce dormancy. The stromal-derived factor 1 (SDF-1, namely CXCL12), and its chemokine receptor CXCR4 play a critical role in PCa bone metastasis. CXCL12, which is produced by osteoblasts in the bone microenvironment, attracts CSCs into the bone environment and occupy the hematopoietic stem cell (HSC) niche 69, 70. Furthermore, overexpression of CXCR4 was positively correlated with cell stemness in hTERT-immortalized human prostate epithelial cells [71, 72.] In addition, the CXCL12/CXCR4 axis plays an important role in attracting HSCs to bone, and maintain a niche for HSCs 73,74. The process is similar to that of CSCs, which are transported to the niche via appropriate CXCL12 and ANXA2 signaling, suggesting that CSCs occupy HSC niche when PCa metastasizes to bone. Once in the niche, the CSCs remain dormant for long periods and interact with the bone microenvironment. The disseminated tumor cells (DTCs) occupying the niche of HSCs, and then the DTCs are induced to CSCs via interactions with the niche. HSCs play a key role in maintaining the stem cell function. The stem cell-related genes such as KLF4, Bmi-1 and Nanog are overexpressed in the DTCs after their arrival at HSCs niche. MicroRNAs (miRNAs) play an important role in regulating various genes, and have become a focus of study in recent years. The miR-141 was found to suppress PCSCs properties in PC-3 and DU145 PCa cell lines, and affected PCa metastasis in an orthotopic cancer model 75. miR-34a was also shown to suppress the function of PCSCs and inhibit cancer metastasis 76,77.

The CSCs and bone marrow microenvironment are closely linked, and signals from bone marrow are very important for the function of CSCs. We will briefly elaborate the most crucial signaling pathway, including those mediated by Notch, Wnt, Hedgehog, FGF, and TGF 78.

### Notch pathway

Notch is a transmembrane receptor expressed in CSCs that plays an important role in stem cell self-renewal and differentiation, and inhibition of this signaling pathway appears to be a therapeutic strategy for CSCs elimination 79. Osteoblasts in the bone microenvironment express the ligand for Notch, and activate the Notch signaling pathway in the CSCs. Notch signaling pathway promotes the EMT process of CSCs and enhances their metastatic ability. It was reported that Notch regulates the EMT process through Snail protein. Notch1 expression in metastatic lesions is substantially higher than in the primary site. When Notch1 is down-regulated, the EMT properties of CSCs are inhibited, while when Notch1 is overexpressed in PCa cells, their migration and invasion ability is enhanced 80. In docetaxel-resistant PCa cells, a subpopulation of CSC-like cells showed activation of Notch and Hedgehog signaling pathway, while the inhibition of Notch signaling reversed the drug resistance 81. Another study showed that pharmacological inhibition of Notch signaling pathway, using γ-secretase inhibitor (GSI), increased the efficiency of castration therapy 82. In yet another study, it was shown that GSI enhanced the antitumor effects of docetaxel in PCa, which might be related to a decrease in PCSCs 83. It was also reported that miRNAs are closely related to Notch signaling in PCSCs. Overexpression of miR-34a in PCa cells reduced Notch1 expression, and decreased the growth and self-renewal of these cells 84. Overexpression of miR-199-5p downregulated the CSC-associated markers in PCa cells and inhibited the Notch signaling of PCSCs 85. One study showed that the CD54-p38-Notch1 axis plays important roles in colony formation, apoptosis, and cancer recurrence 86.

### Wnt pathway

Wnt signaling pathway is very important for embryonic development and adult tissue homeostasis. The canonical Wnt signaling pathway also plays an important role in stem cell self-renewal, maintenance, and differentiation, partly via enhancement of human telomerase reverse transcriptase (hTERT) activity 78,91. Wnt signaling in PCa bone metastasis can enhance osteoblastic activity in the bone microenvironment. PCa cells interact with osteocyte via Wnt signaling to induce differentiation of pre-osteoblasts into mature osteoblasts, while inhibiting the RANKL/OPG signaling pathway to suppress bone resorption 87. Furthermore, the activation of Wnt3 signaling pathway increased the expression of CSC markers, such as CD133, CD44, keratin 18, and β-catenin 88. Specifically, it was demonstrated that the regulation of Wnt pathway altered the CSCs phenotype. For example, miR-320 can alter CSCs phenotype by downregulating Wnt pathway 89, as Wnt pathway regulates the self-renewal and symmetric cell division of PCSCs. Overexpression of PHF21b activates Wnt signaling pathway, leading to an increase in PCSCs traits. Such overexpression is associated with poor prognosis of PCa 90. Many Wnt signaling inhibitors were tested in clinical trials as they decrease the function of CSCs. Most of these inhibitors are fusion-protein, such as the frizzled 8 receptor (FZD8) that is fused to IgG1 Fc fragment 92. FZD8 is an important protein, which is upregulated and positively correlated with PCa progression and bone metastasis clinically 93. Wnt signaling occurs in FZD8-mediated increase in stem cell phenotype, cell migration, and invasion in PCa cell lines 94. Activation of Wnt signaling pathway requires the corresponding ligands, including WNT2B, WNT3, and canonical Wnt ligands to bind with Wnt receptors, leading to suppression of β-catenin, resistance to degradation by GSK protease, resulting in transcriptional activation of target genes, such as *ATOH1*, *CCND1*, *CD44*, *LGR5* and *Snail* 95. Recently, *DAB2IP* was identified as a new tumor suppressor gene in PCa. It regulates the stemness of PCSCs through modulation of CD117 transcription, in which the Wnt/β-catenin signaling pathway plays an important role 96. *DAB2IP* also inhibits the EMT and PCa bone metastasis 97, 98. However, SOX9 was found to regulate Wnt signaling, enhance the EMT process in PCa, resulting in PCa metastasis 99. The miRNAs play an important role in the regulation of CSCs. For example, miR-1301-3p promotes the expansion of PCSCs via activation of Wnt/β-catenin signaling pathway. Prostate CSC-related genes, such as *OCT4*, *SOX2*, *NANOG*, *CD44*, *KLF4*, *c-MYC*, and *MMP2*, are upregulated by miR-1301-3p, while *GSK3β* and *SFRP1* genes are downregulated 100.

### Hedgehog pathway

Hedgehog (Hh) signaling pathway is highly conserved. It plays an important role in embryonic development, stem cells maintenance, and tissue regeneration 101. Abnormal Hh signaling in PCa leads to enhanced progression, relapse, metastasis, and drug resistance 102,103. Recently, several studies have shown that Hh signaling pathway can maintain CSC phenotype. This pathway includes several important components such as Hh ligands, transmembrane receptors PTCH1 and PTCH2, G-protein-coupled receptors such as Smoothened (SMO), and glioma-associated oncogene transcription factors 1 to 3 (Gli1, Gli2, and Gli3) 104,105. When the Hh ligand binds to a PTCH receptor, the signaling pathway is activated, and a Gli transcription factor is translocated to the nucleus, where it promotes target gene expression 106-108. In PCa, the Hh ligands are often synthesized in an autocrine or paracrine manner. For example, bone marrow stromal cells (BMSCs) express Hh ligands and maintain the stemness of PCSCs. Hedgehog signaling also induce the expression of CSC markers, such as BMI1, WNT2, and CD44.

Hh signaling pathway is critically important for CSC functions, and therefore strategies to inhibit the pathway using pharmacological or genetic methods were studied. Cyclopamine, vismodegib, saridegib, and sonidegib are potent and specific SMO inhibitors, with clinical trials indicating therapeutic benefit for patients diagnosed with cancer 109-113. Sonidegib was found to decrease CSC markers, including NANOG, OCT4, SOX2, and c-MYC 114. And Gli inhibitors, such as GANT61, also inhibit PCa growth and proliferation 115. A recent study showed that inhibition of Hh signaling pathway increases the anti-tumor effects of paclitaxel in PCa 116. Perhaps SHH signaling inhibition of PCSCs represents a therapeutic strategy in patients with prostate cancer bone metastasis.

## Dynamic evolution of prostate cancer stem cells in bone metastasis

In fact, the interaction between PCSCs and bone microenvironment is a dynamic process that drives CSC evolution. Rapid division of PCa cells leads to a hypoxic environment, which affects the PCSCs function by enhance their stemness and self-renewal 117.

Disseminated PCa cells can be converted to CSCs in the bone environment, depending on the supply of cytokines and growth factors in the stem cell niches. The CSC phenotype in the bone metastasis is usually defined by the dual expression of CD133 and CD44 118. GAS6, expressed by osteoblast, plays an important role in converting non-CSCs into CSCs through the mTOR signaling pathway. PCSCs in the bone marrow of mice with GAS6 knockout showed a significant decrease compared with wildtype GAS6 mice 119. The downstream signaling pathway of mTOR also plays a critical role in maintaining the CSC phenotype. This signaling is mediated by the Mer receptor on PCa cells. This GAS6/Mer/mTOR pathway reflects the interaction between osteoblast and CSCs in the bone metastatic site. Collectively, the fate of prostate cancer bone metastatic CSCs mainly depends on the bone environment.

Stemness of CSCs is not stable and changes dynamically. It is a transient state that is influenced by genetic, epigenetic, and bone microenvironment. The characteristics of CSCs are affected by their interactions with the bone microenvironment, while factors released by them affect stromal cells in the microenvironment, hijacking them to promote tumor growth. Stromal cells include fibroblast, endothelial, and immune cells, as well as other cell types. The extracellular matrix produced by the stromal cells can also influence CSC function 120,121. Cancer-associated fibroblasts (CAFs) are an important component of cancer stromal cells and are associated with oxidative stress in the microenvironment, with potential for inducing cancer transformation. CAFs induce metabolic changes such as oxidative phosphorylation in cancer cells 122,123. CAFs were shown to regulate the proliferative capability of CSCs in a *PTEN*-gene knockout PCa mouse model. They also activate Wnt and Notch signaling pathways in CSCs to maintain their stemness 124. CAFs can induce EMT and stemness of PCSCs via production of MMPs and activation of pro-inflammatory signaling factors such as NF-κB and HIF.

Recent studies reported that the hypoxic cancer environment plays an important role in CSC evolution. Hypoxia was found to be conducive to establish an acidic tumor microenvironment and activate several proteases facilitating prostate cancer metastasis. The hypoxic environment can induce cancer cells, including CSCs, to express HIF, which enables cellular adaptation to hypoxia 125,126. Further, several studies have shown that hypoxia and HIF induce cancer cells to express stem cells-like phenotype by upregulating the expression of OCT4, SOX2, and NANOG 127,128. The hypoxic environment also activates Notch signaling pathway, thus increasing the population of CSCs and regulating their self-renewal and differentiation. Therefore, when the hypoxic conditions are eliminated, CSCs can be more easily depleted and is conducive for prostate cancer bone metastasis. Evolution of PCSCs is a complex process, affected by various factors that will always occur.

## Dormancy of PCSCs

The dormant state of PCSCs can be induced under a foreign environment of the bone marrow. The dormant state can be maintained for prolonged durations, and might be related to their anti-apoptotic and DNA repair capability 129. When PCSCs enter the dormant state, they become non-proliferative (G_0_ phase of cell cycle), and are highly conserved. In this state, they can bypass traditional therapies. Actually, cell quiescence is closely related to cell dormancy, and CSCs usually utilize these characteristics to evade harsh environment stimuli 130,131. This is not, however, a passive state, but rather an active one that requires various signaling pathways. Factors such as p53, RB, p21, p27, and various miRNAs regulate this process. The dormancy of PCSCs is also supported by the CAFs in the tumor hypoxic environment 132,133. Moreover, disseminated PCa cells in the bone microenvironment also exist in a dormant state for a long time. As dormancy is an important feature of PCSCs, some studies showed that mitotic quiescence rather than surface phenotype can be used to accurately identify PCSCs 134. The PCSCs exit the dormant state in the presence of external factors so PCSCs switch between dormant and proliferative state.

The HSC niche plays an important role in inducing HSCs dormancy. When the PCSCs arrive to the bone marrow, they reside in the HSCs niche and become dormant 135. Inactivation of oncogenes, such as Myc, or activation of p38 stress signaling can contribute to tumor dormancy 136,137. c-MYC is a crucial cell cycle regulator, and its inactivation induce p21 and p27 protein accumulation and dormancy in the cells. Activation of Myc can maintain the stemness of PCSCs, while inactivation of Myc can inhibit PCSCs tumorigenicity. TANK binding kinase 1 (TBK1) plays an important role in maintaining the stemness of PCSCs and inducing dormancy in CSCs. Knockdown of the TBK1 using short hairpin RNA (shRNA) led to activation of mTOR signaling and a decrease in CSCs, which suggests that mTOR signaling pathway is very important for tumor dormancy 138. The PI3K signaling pathway was also shown to be related to the dormant state of PCSCs and inhibition of the PI3K signaling can reduce the PCSCs.

Dormant PCSCs are also an important factor contributing to treatment failure. During therapeutic intervention, the PCSCs often become dormant to evade death induced by anti-proliferating agents, such as docetaxel. Therefore, new strategies should be developed to target the dormant PCSCs, which is a challenge. Strategies that redirect the dormant PCSCs to enter the G_1_ phase of the cell cycle might be an effective approach for future investigation.

## Treatment resistance related to cancer stem cells

Traditional treatment can effectively destroy the non-stem cells cancer cells but not the dormant CSCs 139,140. Current therapies mainly target the fast-growing, differentiated cells, but they do not affect the relatively slow-growing CSCs. The CSCs also express high levels of ATP-binding cassette (ABC) transporters that can induce active efflux of drugs and prevent drug uptake. In such cases, P-glycoprotein, and multidrug resistance associated proteins 1 and 2 (MRP1 and MRP2) are usually overexpressed in CSCs 141-144. Moreover, PCSCs exhibit potent anti-apoptotic capacity, with anti-apoptotic genes such as *Bcl-2*, *Bcl-xl*, and *survivin* overexpressed in some CSCs. These cells have active DNA damage detection and repair systems. Further, PCSCs were shown to be resistant to radiation therapy, which might be related to activation of Chk1 and Chk2 145. Treatment of prostate cancer bone metastases with radiation induced reactive oxygen species (ROS) generation in cancer cells, leading to cancer cell death. However, when the PCSCs were treated with radiation, only limited levels ROS were induced resulting in reduced DNA damage 146-148. Castration is an important treatment method for PCa. However, the cancer cell becomes castration-resistant following prolonged androgen deprivation. Prostate CSCs might play a role in castration-resistance, in which cancer cells show self-renewal and tumor propagation in the absence of androgen receptors 149,150. Growing evidence shows that CSCs surface markers, including CXCR4 and EpCAM, are involved in chemotherapy resistance. Inhibition of CXCR4 by AMD3100 led to enhanced chemotherapeutic efficiency of docetaxel 151. Knockdown of EpCAM by short interfering RNA (siRNA) in PCa cell lines increased their chemosensitivity 152.

Androgen receptor (AR) is very important for the survival of normal prostate cells and prostate cancer cells. In primary and untreated PCa, the PCSCs are AR^-^ and therefore do not respond well to androgen deprivation therapy (ADT). Isolated CD44^+^/ integrin α2β1^+^/CD133^+^ CSCs from several human PCa samples expressed ABCG2, but AR expression was almost undetectable 153. Conversely, loss of AR contributes to PCSCs generation, which might explain the substantially higher levels of PCSCs in castration-resistant PCa than in androgen-sensitive cancers. ADT induces the activation of HIFs, which then lead to PSCSs proliferation and differentiation. ADT downregulates the vascular endothelial growth factor (VEGF) expression, which in turn impairs the tumor vasculature, and thereby induces the hypoxic tumor microenvironment. Both AR^+^ and AR^-^ clones exist in CRPCs. Most PCSCs do not express AR, while some do, and both clones exhibit distinct biological features 154. Conversely, the PCSCs also contributed to ADT resistance.

It is evident that cancer cells are driven and maintained by a set of stem cells. Treatment strategies should be directed to CSCs, which are proposed to be the root cause of cancer. A study of PCSCs showed that 5-lipoxygenase inhibition induced apoptosis of PCSCs by activating c-jun N-terminal kinase and downregulating their stemness 155. The dormant state of PCSCs is also an important factor underlying drug resistance. Strategies for identifying dormant CSCs will lead to therapies targeted at this subclone population of cancer.

Novel treatment strategies and assay systems should be adapted to effectively identify the CSCs, such as those expressing high levels of CD44. When miR-34a was used to inhibit the expression of CD44 in PCSCs, the progression and metastasis of the PCa were remarkably reduced.

## Remaining questions and future direction

PCSCs have gained increasing attention in recent years and are supposed to play an important role in prostate cancer bone metastasis and drug resistance. PCSCs are believed to the precursor of bone metastasis. It is proposed that PCSCs occupy the HSCs niche and remain dormant for extended duration, followed by activation by some signals resulting in bone metastasis. The origin of PCSCs needs to be further studied. How to identify the PCSCs in the samples of bone prostate cancer bone metastasis, and how to accurately discriminate the dormant PCSCs using the proper molecular biology techniques are two important challenges that we are faced. Additionally, the gene instability of PCSCs is closely related to drug resistance. Therefore, the in-depth research for the mechanism of PCSCs gene instability will shed some light on prostate cancer bone metastasis.

## Conclusion

The importance of CSCs in cancer initiation, progression, recurrence, and metastasis is gaining increasing attention. We have discussed here a number of important aspects of PCa: 1) the role of CSCs in PCa bone metastasis and their drug resistance; 2) the complex interactions between PCSCs and the bone microenvironment following bone metastasis of PCa; 3) relevant signaling pathways related to this interaction; 4) the dynamic evolution of PCSCs in the bone microenvironment, as this is a dynamic rather than static process; 5) the dormant state of PCSCs that is vital for the successful formation of bone metastasis; 6) the role of PCSCs in drug resistance, including castration resistance. We hope that this review will advance our understanding of bone metastasis and drug resistance of PCa.

## Figures and Tables

**Figure 1:**
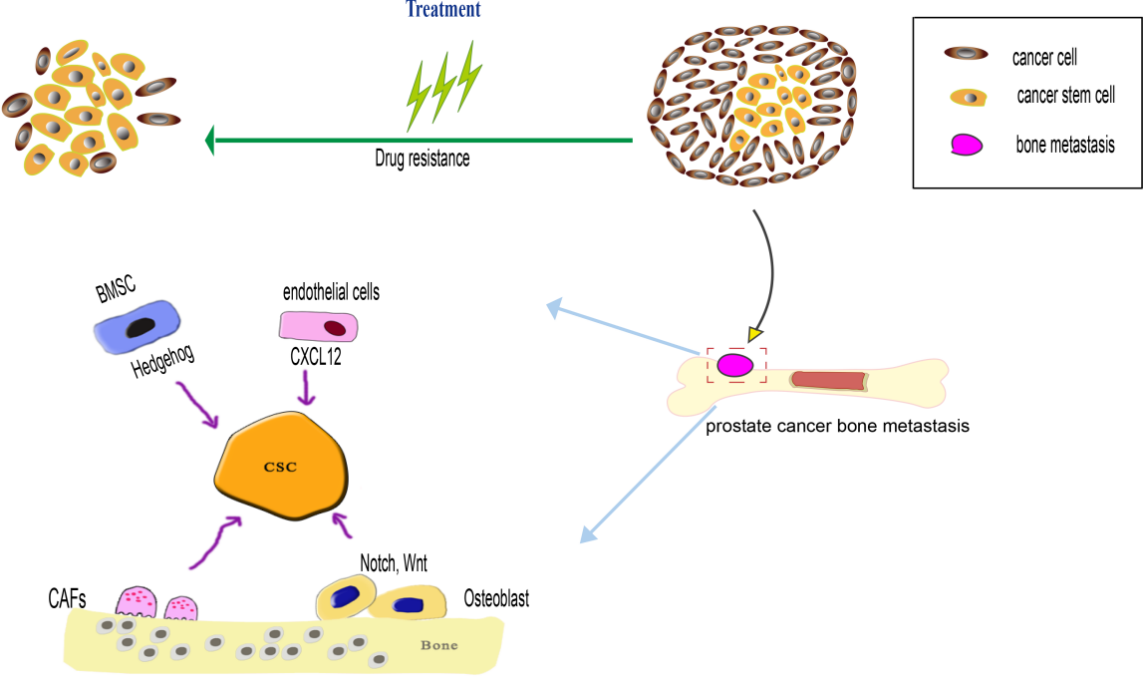
Bone microenvironment factors regulate prostate cancer stem cells (PCSCs) during both processes of prostate cancer bone metastasis and drug resistance. When the PCSCs occupy the hematopoietic stem cells (HSCs) niche, the PCSCs interact with bone marrow stromal cells (BMSCs) and osteoblast, BMSCs influence the PCSCs via SHH signaling pathway, osteoblast influence the PCSCs via Notch and Wnt signaling pathway. The endothelial in the bone marrow microenvironment can secrete CXCL12 to induce PCSCs migration and colonization at the metastatic niche. Cancer-associated fibroblasts (CAFs) can maintain the stemness of PCSCs through MMP secretion and pro-inflammatory signaling pathway. As the PCSCs possess various drug-resistance mechanism, such as overexpression of drug-resistance protein, anti-apoptotic capacity to overcome apoptosis, a dormant state is beneficial to prevent drug-induced death.
